# “Bet hedging” against climate change in developing and adult animals: roles for stochastic gene expression, phenotypic plasticity, epigenetic inheritance and adaptation

**DOI:** 10.3389/fphys.2023.1245875

**Published:** 2023-10-06

**Authors:** Warren W. Burggren, Jose Fernando Mendez-Sanchez

**Affiliations:** ^1^ Developmental Integrative Biology Group, Department of Biological Sciences, University of North Texas, Denton, TX, United States; ^2^ Laboratorio de Ecofisiología Animal, Departamento de Biología, Facultad de Ciencias, Universidad Autónoma del Estado de México, Toluca, Mexico

**Keywords:** climate change, weather, phenotypic plasticity, development, stochasticity

## Abstract

Animals from embryos to adults experiencing stress from climate change have numerous mechanisms available for enhancing their long-term survival. In this review we consider these options, and how viable they are in a world increasingly experiencing extreme weather associated with climate change. A deeply understood mechanism involves natural selection, leading to evolution of new adaptations that help cope with extreme and stochastic weather events associated with climate change. While potentially effective at staving off environmental challenges, such adaptations typically occur very slowly and incrementally over evolutionary time. Consequently, adaptation through natural selection is in most instances regarded as too slow to aid survival in rapidly changing environments, especially when considering the stochastic nature of extreme weather events associated with climate change. Alternative mechanisms operating in a much shorter time frame than adaptation involve the rapid creation of alternate phenotypes within a life cycle or a few generations. Stochastic gene expression creates multiple phenotypes from the same genotype even in the absence of environmental cues. In contrast, other mechanisms for phenotype change that are externally driven by environmental clues include well-understood developmental phenotypic plasticity (variation, flexibility), which can enable rapid, within-generation changes. Increasingly appreciated are epigenetic influences during development leading to rapid phenotypic changes that can also immediately be very widespread throughout a population, rather than confined to a few individuals as in the case of favorable gene mutations. Such epigenetically-induced phenotypic plasticity can arise rapidly in response to stressors within a generation or across a few generations and just as rapidly be “sunsetted” when the stressor dissipates, providing some capability to withstand environmental stressors emerging from climate change. Importantly, survival mechanisms resulting from adaptations and developmental phenotypic plasticity are not necessarily mutually exclusive, allowing for classic “bet hedging”. Thus, the appearance of multiple phenotypes within a single population provides for a phenotype potentially optimal for some future environment. This enhances survival during stochastic extreme weather events associated with climate change. Finally, we end with recommendations for future physiological experiments, recommending in particular that experiments investigating phenotypic flexibility adopt more realistic protocols that reflect the stochastic nature of weather.

## 1 Introduction: climate change, development, and physiology

**FIGURE 1:**
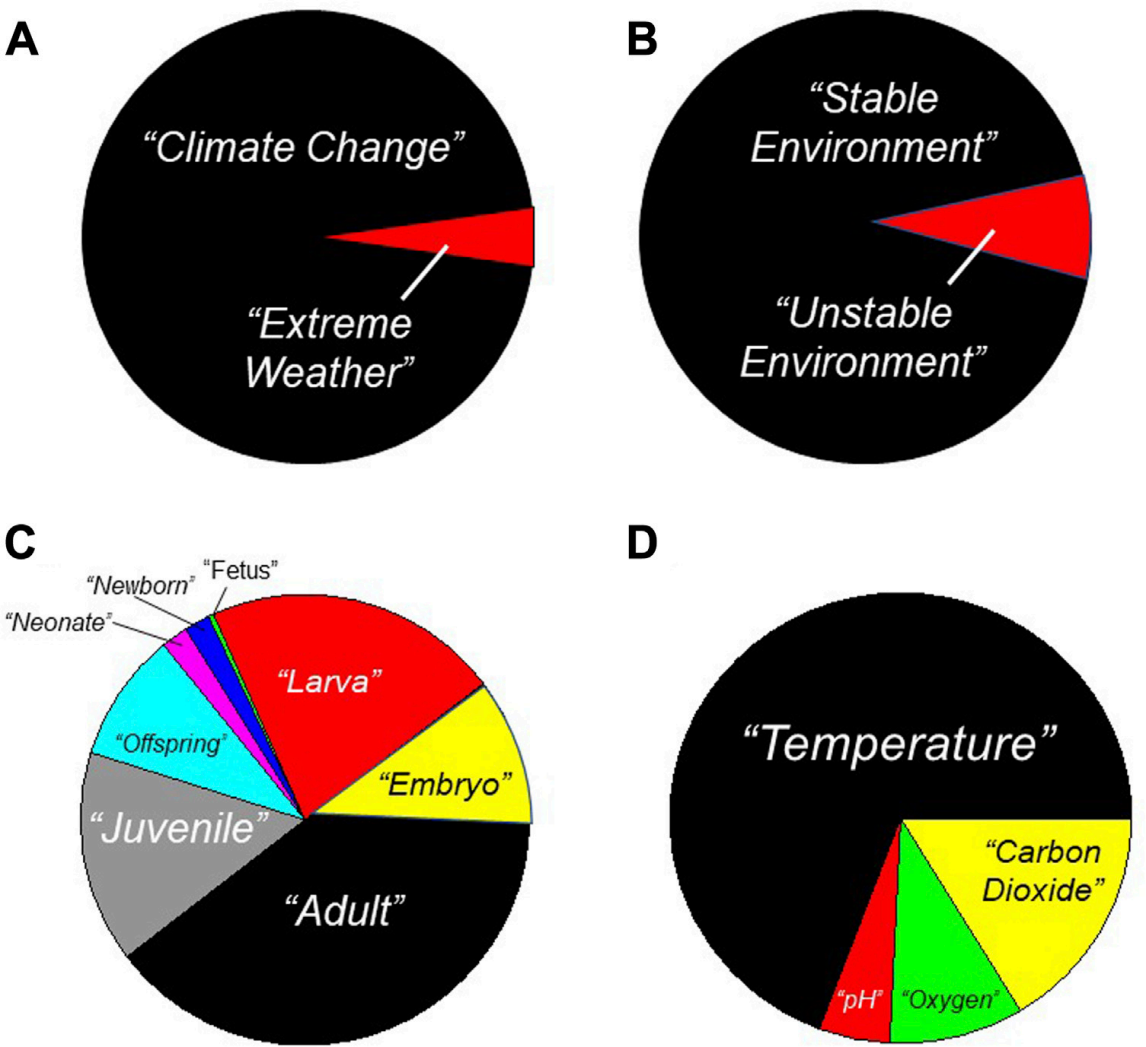
Research focus on Physiology, Climate Change and Developmental Physiology. Results are shown for an August 2023 search of the PubMed data base (https://pubmed.ncbi.nlm.nih.gov/). **(A)** Search terms = “*climate change*” and “*extreme weather*”. **(B)** Search terms = “*stable environment*” and “*unstable environment*”. **(C)** Search terms = “*physiology*” and “*climate change*” and indicated developmental category such as “*larva*”. **(D)** Search terms = “*physiology*” and “*climate change*” and “*development*” and indicated environmental variable such as “*temperature*”. While only very generally indicative of the papers in the PubMed data base, these searches indicate that the great preponderance of studies focus on the effects of incremental (non-fluctuating) temperatures associated with climate change.

Considerable effort is being devoted to investigations of the impact of climate change on organismal physiology. Many of these investigations are focused on the potential for physiological responses to mitigate the predicted effects of climate change. To determine trends in relevant research, in August 2023 we used selected key words to search the PubMed data base (Figure 1). Firstly, this search revealed that >95% of research focuses on climate change rather than extreme weather (Figure 1A). Related to this, >90% of research focuses on stable environments, not unstable environments (Figure 1B). Here, we can loosely equate “extreme weather” with “unstable environment”, and infer that studies on variable environments associated with extreme weather are being greatly eclipsed in number by climate change studies. Secondly, simply using “*physiology”* and “*climate change”*’ as key words reveals that investigation of the impact of climate change specifically on developing animals features prominently (Figure 1C). This focus on development is likely because there has long been awareness of how environmental stressors and toxicants frequently exert a disproportionate effect during development, which is replete with critical windows (‘sensitive periods’) (Hensch and Bilimoria, 2012; Wells, 2014; Burggren and Mueller, 2015; Tate et al., 2015; Mueller, 2018; Andrade-Talavera et al., 2023). Indeed, using the search terms indicated in the legend to Figure 1C, more than half of the PubMed-identified studies investigating climate change and associated physiological responses mentioned specific developmental stages–e.g., ‘larva’, ‘neonate’, *etc.* Predictably, given the focus of climate change researchers, nearly three-quarters of these studies incorporated physiological study of temperature effects related to climate change (Figure 1D). A little less than one-third of these studies were devoted to climate change-related water acidification effects as well as the effects of decreased oxygen and increased carbon dioxide–e.g., (Mukherjee et al., 2013; Jackson et al., 2016; Hannan and Rummer, 2018; Teawkul and Hwang, 2018; Watson et al., 2018; Bairos-Novak et al., 2021; Chille et al., 2022; Czaja et al., 2023; Glass et al., 2023).

This focus on the physiological effects on developing animals associated with predicted long-term changes in environmental variables such as temperature will doubtlessly prove necessary for our understanding of the biological implications of climate change. In this review we hypothesize that understanding the effects of especially short-term environmental fluctuations on developing animals will be a key part of revealing the full impact of global climate change and understanding to what extent organisms can survive current and future environmental stressors. To this end, we also consider the concept of ‘bet hedging’ and fluctuating environments–namely, that different phenotypes in a population will have varying degrees of fitness to any specific environment, potentially lowering the overall population fitness but ensuring the existence of some individuals favorably adapted to an altered environment.

## 2 Climate change–The challenge

### 2.1 Differentiating weather from climate

Most biological studies of future effects of environment change–certainly studies in the area of developmental physiology–are focusing on climate change*.* However, ‘climate change’ is variously defined, and its interpretation has different implications for different ecosystems and the developing organisms within them. Consider, for example, the oceans, which are predicted to experience a slow but inexorable rise in temperature and fall in pH as global climate change progresses. The sheer mass of the oceans and their relative heterogeneity is such that researchers can reasonably focus on changes in ‘climate’ designed around relatively predictable, steady change across the years, decades and centuries. Contrast this with small freshwater ecosystems or terrestrial environments where temperatures, for example, can vary enormously over a single season or even few days (Collins et al., 2013). The predicted *average* global temperature increase of 2–4°C by the year 2,100 (Collins et al., 2013)—what we might call ‘climate’ - has diminished meaning when designing experiments investigating the effects on phenotype of temperature change in rapidly changing situations–that is, ‘weather’. Indeed, numerous individuals over the last century and a half have offered up variations on the aphorism that *climate is what we expect, while weather is what we get* (Twain, 1871; Herbertson, 1901; Heinlein, 1973). The seeds of global climate change were yet to be sown when these early authors clearly distinguished the capricious nature of ‘weather’ from the more stable ‘climate’. Why does this aphorism hold such significance for developmental physiologists studying phenotypic plasticity?

### 2.2 weather-vs. climate-related experiments and their implications

Large temporal and spatial differences in environmental conditions are likely to accelerate as global warming continues (Collins et al., 2013). We suggest that short-term weather-driven environmental change for species- and population-level survival can actually be quite profound. A species may well be capable of resisting a predicted *climate* change of, for example, a 5°C increase in environmental temperature over decades or centuries. However, on the way to that long-term predicted change, populations of that species may experience numerous increasingly extreme *weather* events that could produce much higher (or lower) temperature excursions that fall outside the survivable temperature range. Not only do the absolute high and low temperatures experienced represent potential extinction events, but so, too, do the rates of temperature change, which in of themselves can be extreme (Burt, 2007). As a few examples, in 2018 Saranac Lake in upstate New York, United States experienced a temperature drop of ∼47°C in 36 h, while in 2018 Oklahoma City, OK, United States recorded a temperature drop of 12°C in just 240 s (Burt, 2018)! Temperature rises can also be rapid, with Loma, MT, United States experiencing a 48°C temperature rise in just 23 h in 1972. While these are extreme examples of temperature shifts, there is little doubt that extreme weather events–whether involving temperature, precipitation, wind, *etc.*,—are accelerating (Turner et al., 2020; Williams et al., 2021; Abbass et al., 2022; Rorie, 2022).

We posit that it is these short-term excursions falling under the category of ‘weather’ are likely to be a much greater threat to species and population level survival than predicted long-term changes in climate. Yet, many biological studies addressing climate change are framed around changes in environmental variables associated with predicted long-term changes. These studies frequently pose experimental questions such as “*What happens to my organism if environmental temperature increases five degrees?”* In such studies, typically a wide variety of variables are collected in the domains of morphology, physiology, biochemistry, behavior, *etc.*, and the experimental subjects (usually adults) that are heated are compared to unheated controls to determine the effects of the temperature perturbation. Yet, potentially more ‘realistic’ than laboratory simulations of environmental conditions mimicking the distant future are experiments designed around short-term environmental changes that resemble far less predictable and even chaotic weather events (Saunders et al., 2002; Colinet et al., 2015; Drake et al., 2017; Burggren, 2018; Huey and Buckley, 2022; Ridgway and Scott, 2023). We argue here that such experiments are not only more relevant for many species and many ecosystems, but are likely to lead to more accurate assessment of the ability of species and their populations and individuals to actually favorably respond and even keep pace with climate-induced environmental change.

Before discussing how animals of all developmental stages may cope with climate and, perhaps more urgently, with weather, we briefly review how adaptative phenotypes can emerge.

## 3 Mechanisms for phenotypic change

**FIGURE 2:**
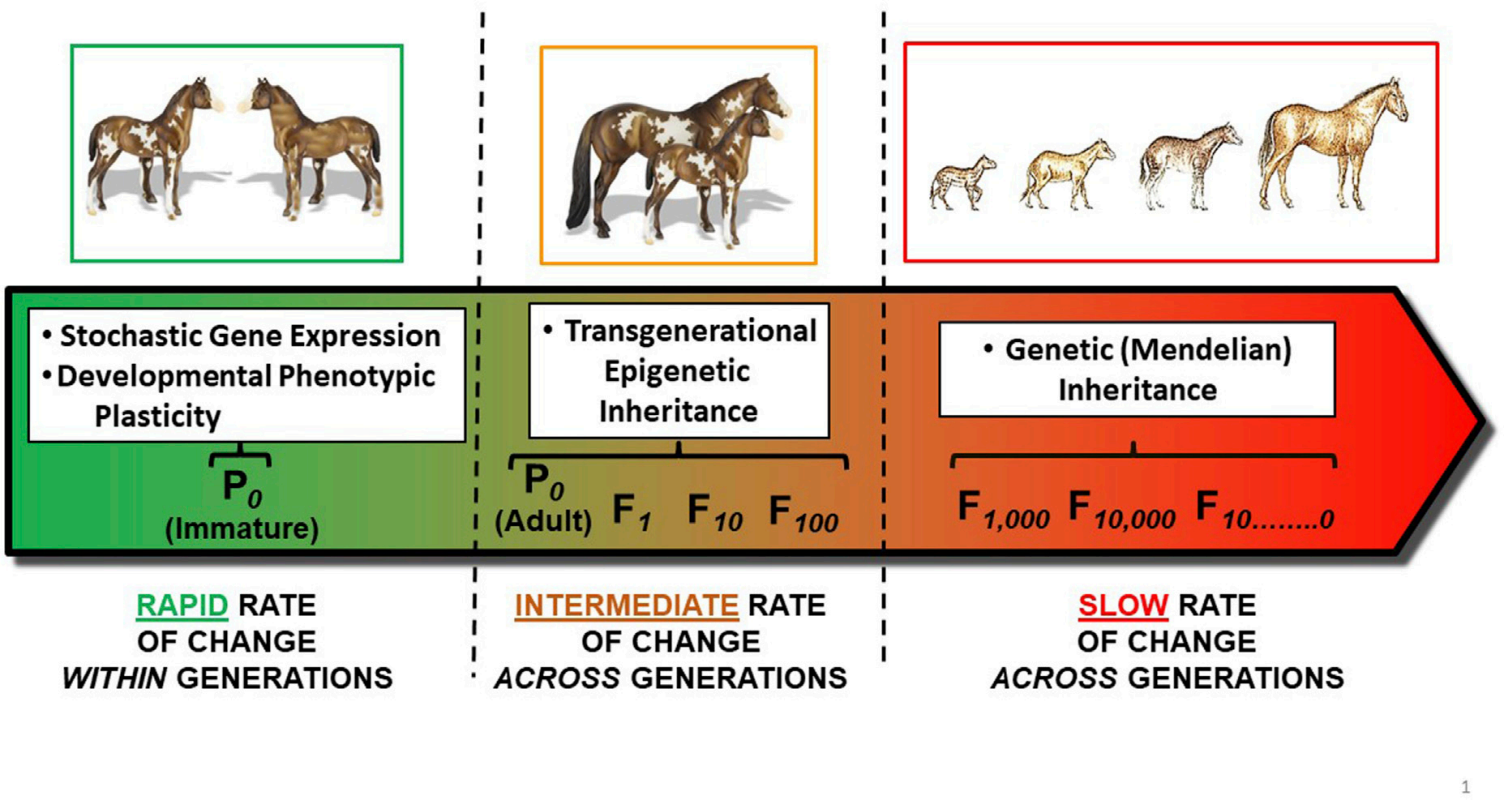
Mechanisms for phenotypic change over time. Left Panel: Developmental Phenotypic Plasticity acts within the span of a single generation. Both stochastic gene expression and developmental phenotypic plasticity during development can create different phenotypes within a population. In this example, each colt has a different dappling pattern, which can also change during further development. Middle Panel: Transgenerational epigenetic inheritance of a modified phenotype results from the actions of so-called readers, writers, eraser and enhancers on the epigenome which, in turn, results in modified gene expression in the F_1_ generation (and potentially additional generations). Here, the dappling pattern of the mother’s colt may reflect changes in gene expression rather than changes in gene sequence. Right Panel: ‘Classic’ Mendelian Inheritance of modified phenotype results from inheritance of a set of alleles typically inherited from both male and female parents (P_0_s) across an evolutionary time scale. This example shows the evolution of height and other features from *Hyracotherium* (dawn horse) to modern day horses (*Equus caballus*) over ∼50 million years.

There are several mechanisms by which an organism’s phenotype (physiological, morphological, behavioral, *etc.*) can be altered. These include stochastic gene expression, phenotypic plasticity (including developmental phenotypic plasticity), epigenetic inheritance, and Mendelian inheritance through natural selection. Each of these mechanisms acts in a very different framework of time. Illustrating this point, (DeLiberto et al., 2022), studying thermal sensitivity in the killifish *Fundulus heteroclitus*, observed that “*Physiological responses are driven by temperature on three time scales: acute, acclimatory and evolutionary*”. Here, we break down these vastly different time scales for responses to temperature by considering underlying mechanisms by which animals might respond to environmental stressors–especially temperature–associated with climate change (Figure 2):

• Stochastic phenotypic variation, occurring from stochastic variation in gene transcription and translation, especially during development, that are not related to environmental cues;
• Phenotypic plasticity operates within the timeframe of an organism’s entire life span, and involves the ability to alter phenotype through acclimatization (acclimation);
• Transgenerational epigenetic inheritance influences phenotype of a species over typically a few generations through changes in gene expression (but not sequence);
• Classic Mendelian inheritance acts over large number of generations–that is, evolutionary time–through permanent changes in gene sequence.

Important to emphasize is that these distinct mechanisms–all potentially effective for responding to environmental change - are not mutually exclusive. Thus, even as an organism in response to an environmental stressor may be evolving adaptations through natural selection over many thousands of generations, it may also be showing phenotypic changes through epigenetic inheritance in the F_1_ generation (and potentially beyond). Moreover, within generations, developmental phenotypic plasticity through differential expression of genes (stochastic or otherwise) can be altering physiology, morphology behavior, *etc.*, of an organism on the pathway to sexual maturity within a single generation.

Differentiating between these mechanisms can be challenging (see Section 3.5). Moreover, each mechanism for producing potentially adaptive (favorable) phenotypes in response to climate change has its own advantages and disadvantages, especially in the context of rapidly changing environmental conditions. Before considering how these mechanisms for phenotypic modification interface with climate change and alter an individual’s, population’s or species’ chances for survival, let us briefly consider each mechanism in turn, especially in the context of speed of action.

### 3.1 Stochastic gene expression

Stochastic gene expression results from variable gene expression from a single genotype in the absence of any environmental cues. This contrasts with phenotypic plasticity, triggered by specific environmental cues such as physico-chemical variables, nutrient availability, predation, *etc.* (see Section *3.2.2.*, below). Many have written about stochastic gene expression, and we refer the reader to several instructive reviews including (McAdams and Arkin, 1999; Kaern et al., 2005; MacNeil and Walhout, 2011; Woods, 2014; Vogt, 2015; Chubb, 2017; Bohrer and Larson, 2021; Voortman and Johnston, 2022). Essentially, stochasticity of processes extends to molecular levels. Thus, for example, both gene transcription (Dattani and Barahona, 2017) and translation (Dastidar and Nair, 2022) will show stochastic elements, resulting in spontaneous changes in protein synthesis that vary from time to time during development in an individual, and between individuals. Stochastic gene expression can wax and wane, depending upon the concentration of transcriptional regulators (McAdams and Arkin, 1999). Recent studies have determined that transcription is a discontinuous process (“transcriptional bursting”) that has been both assessed empirically and modeled (Nicolas et al., 2017; Leyes Porello et al., 2023). Moreover, with the onset of senescence, specific DNA methylation patterns break down, presumably resulting in enhanced stochastic variations in gene expression (Soto-Palma et al., 2022). Collectively, these stochastic changes in processes behind gene expression will create phenotype variation. It has long been appreciated that genetically identical organisms grown in identical environments nonetheless show individual stochastic variation (McAdams and Arkin, 1997). Moreover, as the name suggests, these phenotype variations occur independent of environmental cues. While we might assume that the level of stochastic variation in gene expression and its products would not be correlated with climate change, modeling effects like these is quite complex (Bard, 2022). Indeed, although by definition stochastic gene expression is … stochastic … , it is even possible that the degree of stochastic gene expression itself could vary between individuals whose specific molecular phenotype has been under selective pressure associated with exposure to specific environments during development or as adults (Mora and Walczak, 2013). Certainly, more studies on the how the degree of stochastic gene expression is heritable requires more study.

We turn now to those mechanisms for phenotypic variation that are demonstrably dependent on environmental cues.

### 3.2 Developmental phenotypic modification: variation, plasticity, flexibility, and programing

Understanding developmental plasticity first requires clarification in the use in the literature of the terms ‘phenotypic variation’, ‘phenotypic plasticity’, and ‘phenotypic flexibility’ (Piersma and Drent, 2003; West-Eberhard, 2003; Kelly et al., 2012; Woods, 2014; Lee et al., 2022). All describe a product of the expression of a gene or a set of genes, and all are subject to natural selection and thus contribute to evolution. Importantly, phenotypic variation, plasticity, and flexibility can occur simultaneously during both predictable and stochastic environmental change.

#### 3.2.1 Phenotypic variation

The measure of the different phenotypes for a trait that are present in a population at the same time in a given ecological niche (Hallgrímsson and Hall, 2005). Examples are abundantly evident by looking at any group of a single species–variations in hair or fur color, body mass, metabolic rate, or behavior. This variation is the manifestation of the population’s genetic variation, where each gene or set of genes are favorably selected when they impart a positive impact on the individual fitness (survival or reproduction). The raw material for evolution through natural selection, phenotypic variation works in generational time where in each generation there is a quantitative increase of the genotypes and phenotypes with a positive impact on the individual fitness, thus driving adaptations (West-Eberhard, 2003; Lee et al., 2022).

#### 3.2.2 Phenotypic plasticity

Phenotypic plasticity underlies the different phenotypes emerging from a single genotype, produced by developmental or physiological responses to environmental stressors (broadly defined) during a single individual’s lifetime. Multiple mechanisms are responsible for phenotypic plasticity, including epigenetic modification of gene expression, post-translational modification and macromolecular interaction, to mention but a few (Burggren and Reyna, 2011; Kelly et al., 2012; Sahoo et al., 2020). Ultimately, phenotypes modified through these mechanisms result from either 1) direct or indirect effects of environmental factors (e.g., elevated temperature, reduced oxygen, increased/decreased pH, nutrient availability, buildup of nitrogenous waste products), where ‘environment’ spans from intracellular to external environments; or 2) stochastic gene expression (see above).

Importantly, phenotypic plasticity is not just a phenomenon in adults, but rather includes cases of fixed, irreversible, and distinct developmental trajectories that may or may not culminate in altered adult phenotype (Kelly et al., 2012; Burggren, 2020). Phenotypic plasticity in its many forms has been documented in detail for adult and developing organisms and for describing individual variation within a population (West-Eberhard, 2003; Pigliucci, 2005; West-Eberhard, 2005; Grantham et al., 2016; Burggren, 2018; Jolly et al., 2018; Pelster and Burggren, 2018; Westneat et al., 2019; Burggren, 2020; Lee et al., 2022).

Phenotypic plasticity can be among the fastest mechanisms for favorable responses to stochastic environmental changes (other than avoidance behavior when stressors are confined to microenvironments). For example, many species of fishes can remodel their gills in response to environmental stressors in the form of changes in ion concentration, pH or hypoxia of the water they are breathing (Sollid and Nilsson, 2006; Nilsson et al., 2012; Gilmour and Perry, 2018; Braz-Mota and Almeida-Val, 2021). Amazingly, in species like the crucian carp (*Carassius*), and goldfish (*Carassius auratus*), these changes in gill structure can begin within hours of experiencing the stressor (Sollid and Nilsson, 2006).

Phenotypic plasticity is considered to be a component of acclimatization/acclimation, and can occur at potentially any point in time during an individual´s life cycle. Ultimately, the time required for an organism’s phenotype to change as a result of plasticity mechanisms is dependent upon a biological sensor detecting the environmental change plus the time to turn on or off a gene or protein expression to create a modified phenotype.

#### 3.2.3 Phenotypic flexibility

Phenotypic flexibility is a component of phenotypic plasticity, but involves rapid, reversible within-individual variation. For example, iterative seasonal reproducers may have a breeding phenotype that minimizes eating and maximizes activity of the reproductive organs. After breeding is completed, this breeding phenotype is replaced by the non-breeding phenotype that allows them to eat to store energy or even hibernate. Breeding and non-breeding animals may alternate these behavioral, physiological, and metabolic reversible phenotypes several times during their lives (Partecke et al., 2004; Nzama et al., 2010; Oliveira et al., 2016; Boratyński et al., 2017; Campobello and Sealy, 2018; Sommers et al., 2019). This form of phenotypic flexibility is evoked by environmental conditions that either vary predictably (e.g., with season) or that fluctuate more stochastically (Bradshaw, 1965; Piersma and Drent, 2003; Piersma and Van Gils, 2011; Kelly et al., 2012).

#### 3.2.4 Developmental Programming

So-called *developmental programming* is a ‘hot topic’ especially in the medical field, with over 13,000 papers published on this topic, with nearly 3,000 of these appearing from 2020 to 2022 (the last year for complete data, source: Pubmed.gov). Developmental programming, also called ‘fetal programming’, essentially describes the influence of perinatal and especially pre-natal factors on subsequent development.

#### 3.2.5 Phenotype, development, and climate change

**FIGURE 3:**
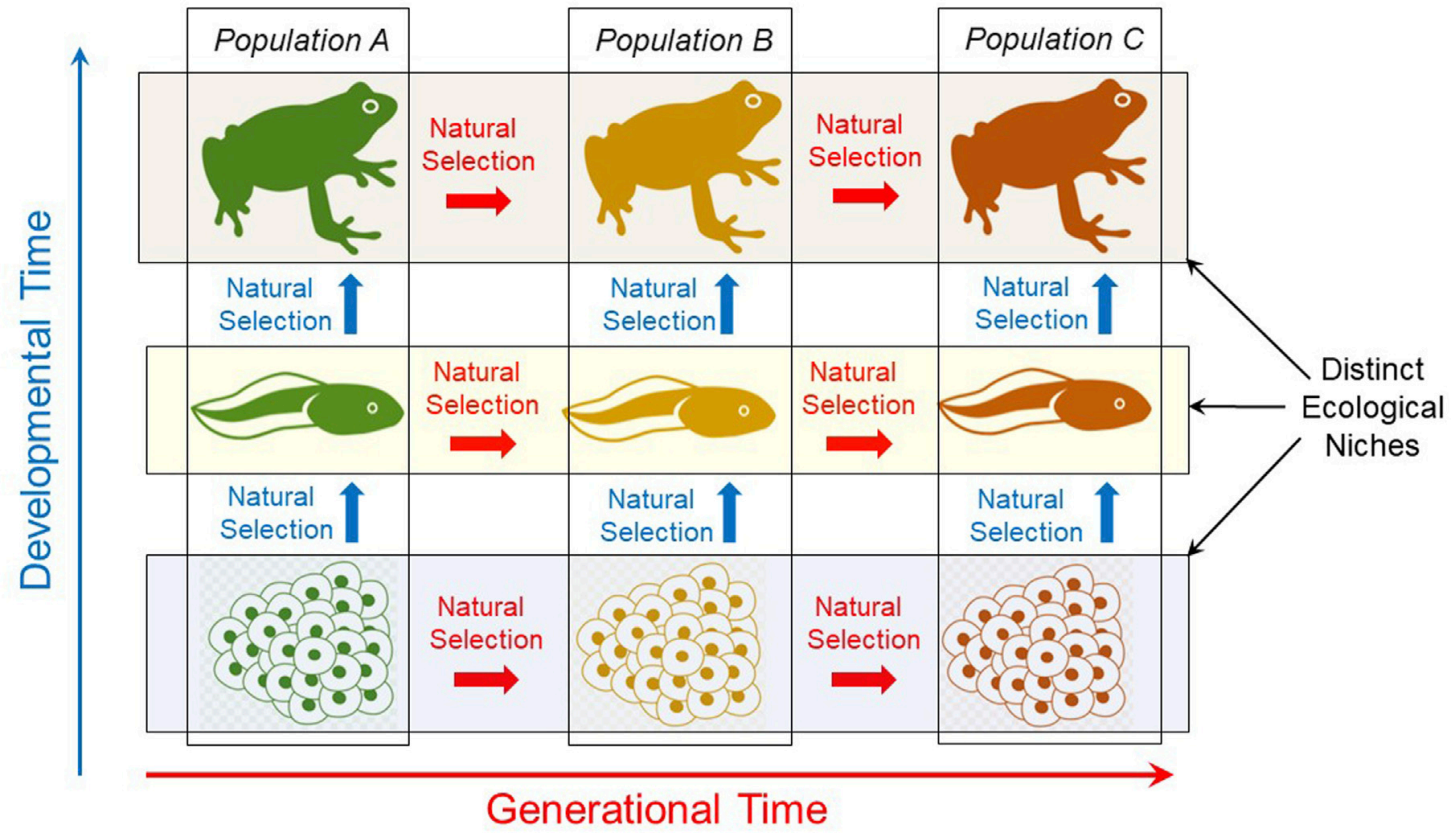
Natural selection occurs throughout ontogeny as well as across generations. Though sometimes ignored, it is important to emphasize that natural selection acts on all developmental stages (vertically arrows), not just adults across generations. Thus, evolution is properly viewed as including change in a series of ontogenies, not just evolutionary change in adults of a population or species across evolutionary time (horizontal arrows).

During development in a fluctuating environment, different genes or sets of genes are variably expressed or remain silent, producing phenotypic variation or flexibility. This variability specifically during development can speed up evolutionary responses to changing environments (Frank, 2011). Adding to the complexity, different genes respond differently at different developmental stages according to temporal patterns of environmental change or specific ecological niche occupied by that developmental stage (Woods, 2014). Thus, at each successive developmental stage, new genes/gene sets will be variably expressed in response to the environment or next ecological niche. For each developmental stage, this environmentally-sensitive phenotype, not the genotype or the gene itself, is experiencing selection and producing individuals with differential fitness that may or may not be suitable for the next ecological niche (West-Eberhard, 2003; West-Eberhard, 2005). The result is a series of physiological developmental phenotypes, highly selected for a series of fluctuating environmental moments or ecological niches. Importantly, the adult phenotype is only the last stage of this phenotypical selected chain. As indicated in Figure 3, natural selection–and thus evolution of a species–occurs across potentially all developmental stages.

Important in a discussion of developmental phenotypic plasticity is consideration of critical windows (susceptible or sensitive periods) for development. These are the periods of time during ontogeny when an organism’s developmental trajectory is altered in response to stochastic environmental factors that requires a rapid response, even outside of their physiological tolerances necessary to stay alive and continue to develop (Pigliucci, 1998; Pigliucci, 1998; Burggren and Reyna, 2011; Burggren and Reyna, 2011; Mendez-Sanchez and Burggren, 2014; Mendez-Sanchez and Burggren, 2014; Burggren, 2018; Burggren, 2018).

**FIGURE 4:**
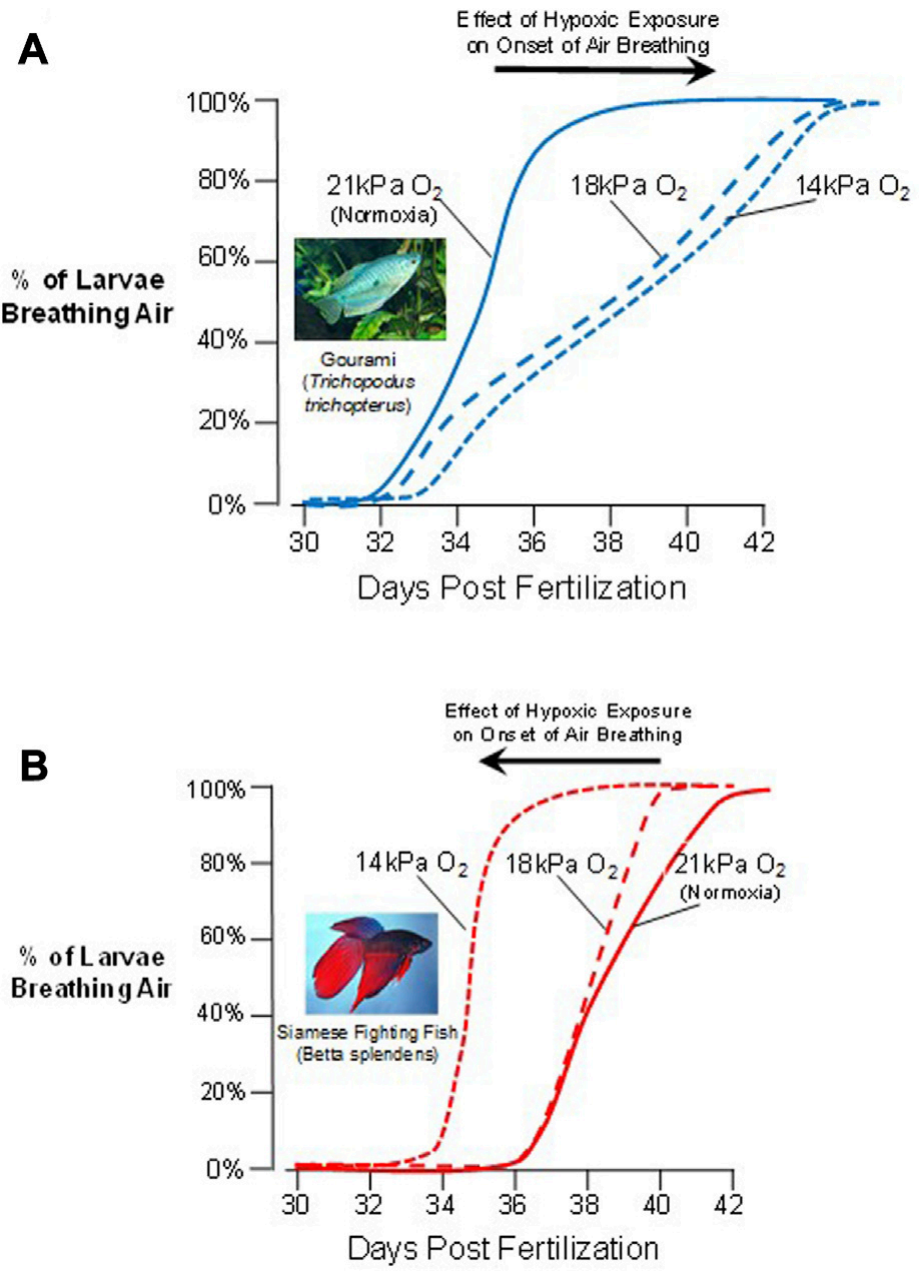
Phenotypic plasticity in the onset of air breathing in two sister-species of labyrinth fishes. **(A)** In response to decreasing levels of aquatic oxygen, the three-spot gourami (*Trichopodus trichopterus*) delays the onset of air breathing. **(B)** In contrast, the Siamese fighting fish (*Betta splendens*) accelerates the onset of air breathing as larvae during varying degrees of hypoxic exposure (Data from Mendez-Sanchez and Burggren (2014)). Larvae of both species thus show developmental phenotypic plasticity, even though the responses to the same stressor (hypoxia) are in opposite directions.

An example of developmental plasticity and flexibility is the environmentally-driven variability in the onset of air breathing (OAB) in air-breathing fishes. This behavior forms a discrete developmental marker that occurs when larval air-breathing fish take their first air gulp, and marks a transition from branchial and cutaneous gas exchange towards even more complex gas exchange with the inclusion of air breathing. Effective air breathing is the culmination of the maturation of an entire suite of structures and processes, including ventilatory control mechanisms, effective blood perfusion pathways, specialized air–blood interfaces that create a functional air-breathing organ and, of course, the behavioral drive to do so (Burggren and Warburton, 2007). Air breathing is also a trait that is likely to confer high fitness on aquatic larval fishes exposed to aquatic hypoxia, and so hypoxia-driven natural selection of a development plan for air breathing that is modifiable through developmental phenotypic plasticity might be anticipated. Against this background, particularly interesting is the OAB in the larvae of two sister-species of labyrinth fishes (Anabantiformes: Osphronemidae) - the three-spot gourami, *Trichopodus trichopterus* and the Siamese fighting fish, *Betta splendens.* Indeed, OAB in these labyrinth fishes is a clear example of fixed and irreversible developmental plasticity, with distinct developmental trajectories set by environments experienced during critical windows. Interestingly, though the species are closely related, the OAB of the gourami and Siamese Fighting Fish showed distinctly different responses to aquatic hypoxia experienced as larvae. In the gourami, OAB occurred at ∼35 dpf in normoxia (21 kPa PO_2_), but severe intermittent nocturnal hypoxia (14 kPa PO_2_) delayed appearance of this developmental maker by several days (Figure 4A). After this point in development, larval gourami become obligate air-breathers, unable as juveniles and adults to extract oxygen solely from the water surrounding them. In stark contrast to the gourami is the phenotypic response to hypoxia of the larvae of the Siamese fighting fish. The OAB of Siamese fighting fish in normoxic exposure was at 39–40 dpf, ∼3 days later than the gourami. However, unlike in the gourami, mild intermittent nocturnal hypoxia (17 kPa PO_2_) actually accelerated (brought forward) the onset of air breathing by ∼4 days and as this species progressively became a facultative air-breather, ultimately being able to extract oxygen from both air and water, an example of developmental flexibility. While tempting to speculate, we do not yet know enough about the interfaces of ecology, environment, development, physiology and morphology in anabantid fishes to interpret these differences in OAB as conferring a selective advantage to one species over another.

### 3.3 Epigenetics and phenotypic change

#### 3.3.1 Epigenetic inheritance

Our understanding of epigenetics and epigenetic inheritance has burgeoned in recent years–for an entry into the literature see (Janssen et al., 2017; Eirin-Lopez and Putnam, 2019; Roberts and Hay, 2019; Sarkies, 2019; Walker and Burggren, 2020a; Safi-Stibler and Gabory, 2020). Modification of the epigenome and its influence on phenotype within an individual’s lifespan has been extensively studied in the context of topics as diverse as preventing/treating human disease (Lin et al., 2023; Paccosi and Proietti-De-Santis, 2023; Sum and Brewer, 2023) to enhancing agricultural production (Feng et al., 2023; Louis et al., 2023; Wang et al., 2023). Here, however, we focus on *transgenerational* epigenetic inheritance. While many have offered both definitions and ‘tests’ for epigenetic inheritance (Berger et al., 2009; Dupont et al., 2009; Pickersgill, 2021; Ospelt, 2022; Švorcová, 2023), we adopt here a broad interpretation of ‘epigenetics’, simply referring to inheritance that does not involve gene modification, but rather modified expression of existing genes (Burggren and Crews, 2014). Surprisingly, given the increasing recognition of the importance of this mechanism for phenotypic change, we still have much to discover about the mechanisms by which the environment and experiences of the P_*0*_ parental generation result in the inheritance of epigenetic markers transferred in the germ line (or possibly through parental provisioning in supporting materials such as cytoplasm or egg albumin and yolk). What is understood is that the epigenome of an organism (and thus the expression of its genes) is in constant flux through the action of a suite of specialized proteins acting as so-called ‘readers’, ‘writers’, ‘erasers’ and ‘enhancers’ (Trevino et al., 2015; Yang et al., 2016; Biswas and Rao, 2018; Walker and Burggren, 2020b), as well as more recently emphasized ‘enhancers’ that can facilitate all of these actions (Wu et al., 2023). By these various related mechanisms for altering gene expression in the F_*1*_ generation, it follows that phenotype will be altered accordingly.

#### 3.3.2 The ‘epigenetic advantage’: fast but transient

**FIGURE 5:**
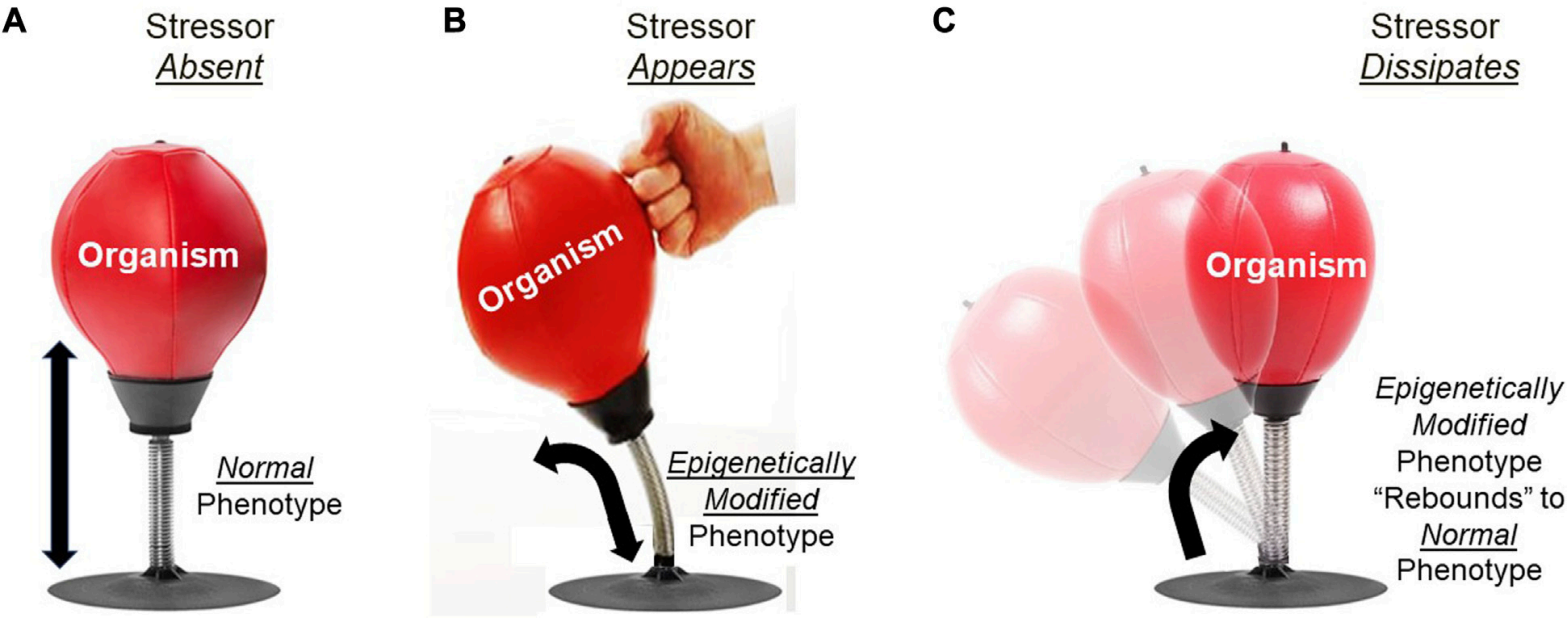
Analogy for understanding rebound of epigenetically-produced phenotypes in variable environments. **(A)** The punching bag on its spring stand (organism) remains in a stable, default configuration (normal phenotype) when it is not being punched (environmental stressor). **(B)** When punched, the bag will assume a new configuration (epigenetically modified phenotype) that will be maintained as long as the punching continues. **(C)** When punching stops (stressor dissipates), the punching bag rebounds to its previous configuration (normal phenotype).

Epigenetic inheritance of a modified phenotype from a parental generation experiencing an altered phenotype is a dynamic process with respect to the time course of its action. Epigenetically inherited phenotypes typically last only a generation or two before “washing out”, resulting in the restoration of the original phenotype in the absence of continuing modification of the genome by the epigenome (Burggren, 2015; Eirin-Lopez and Putnam, 2019). The dynamics of this process can by explained through the analogy of an organism’s phenotype likened to a spring-mounted punching bag (Figure 5). In this analogy, there is an inherently stable, ‘default’ configuration of the punching bag on its spring that occurs in the absence of any punching action. When the bag is punched, it is tilt is altered - but only as long as the punching continues. However–and this is key–if the punching action ceases, the bag immediately springs back to its default, stable configuration. Now, returning to epigenetics, consider the organism as the punching bag being ‘punched’ by the environmental stressor–increased temperature, for example. As long as the heat stress persists, the epigenome responds with altered gene expression leading to a favorably modified phenotype (the tilted punching bag), potentially lasting over multiple generations if the heat stress persists that long. However, if the heat stressor diminishes or especially if it disappears altogether, then the alternative phenotype of the organism will rebound (‘spring back’) to its original configuration–that is, to the default phenotype generated by its genotype lacking abnormal actions of epigenetic readers, writers, erasers and enhancers induced by the heat stress.

Importantly, the influence of an environmental stressor like elevated temperature (or hypoxia, diminished food resources, competition, *etc.*) rarely influences just a single organism in a general population. Rather, most if not all of the population will experience the stressor and consequently most, if not all, will potentially develop a modified phenotype that potentially conveys a tolerance or other favorable modification to the adverse environmental conditions (Figure 5). This results in the phenotype of the entire population simultaneously changing in a similar if not identical direction as a result of the stimulation of the inherent epigenetic mechanism in all individuals of that population. As long as the heat or other stressor persists–potentially across multiple generations - the modified phenotype will be present. However, when the stressor diminishes or disappears, the phenotype of not just the individual but potentially the whole population will “spring back” to the original phenotype (Figure 5). An extreme example of rapid phenotypic change and rebound occurs in the desert locust, *Schistocerca gregaria*, found in Africa, the Middle East and Southeast Asia (Lecoq, 2022). In the absence of rain, individual locusts exist in a behaviorally solitary state, with typically green coloration. However, when a certain set of conditions of temperature, rainfall, animal density and food develops, the solitary phenotype begins to respond by beginning the transition to a morphologically brown, behaviorally gregarious state, that ultimately leads to the infamous ‘biblical swarms’ of locusts. Remarkably, the behavioral changes can occur within 12 h, with the morphological and physiological changes lagging behind by weeks or months. Equally remarkably, the shift from solitarious to gregarious phenotype is now known to be produced by changes in the epigenome triggered by changing environmental conditions (see (Burggren, 2017) for details). Of key importance, it is not just a few individuals that develop a modified phenotype. In fact, as the historically largest swarming events of up to an estimated 10 billion locusts begins to occur (Rainey, 1954) there is a nearly simultaneously initiated epigenetically-driven phenotypic transition in the entire swarm. Equally important, this modification of the epigenome leading to modified gene expression is NOT a permanent change to the general population, as would occur if mutation occurred in the genome. Indeed, with a return of dry conditions (usually within a few generations) this extraordinarily large population typically reverts back to the solitarious green morphological and behavioral phenotype (until the next outbreak of wet weather).

**FIGURE 6:**
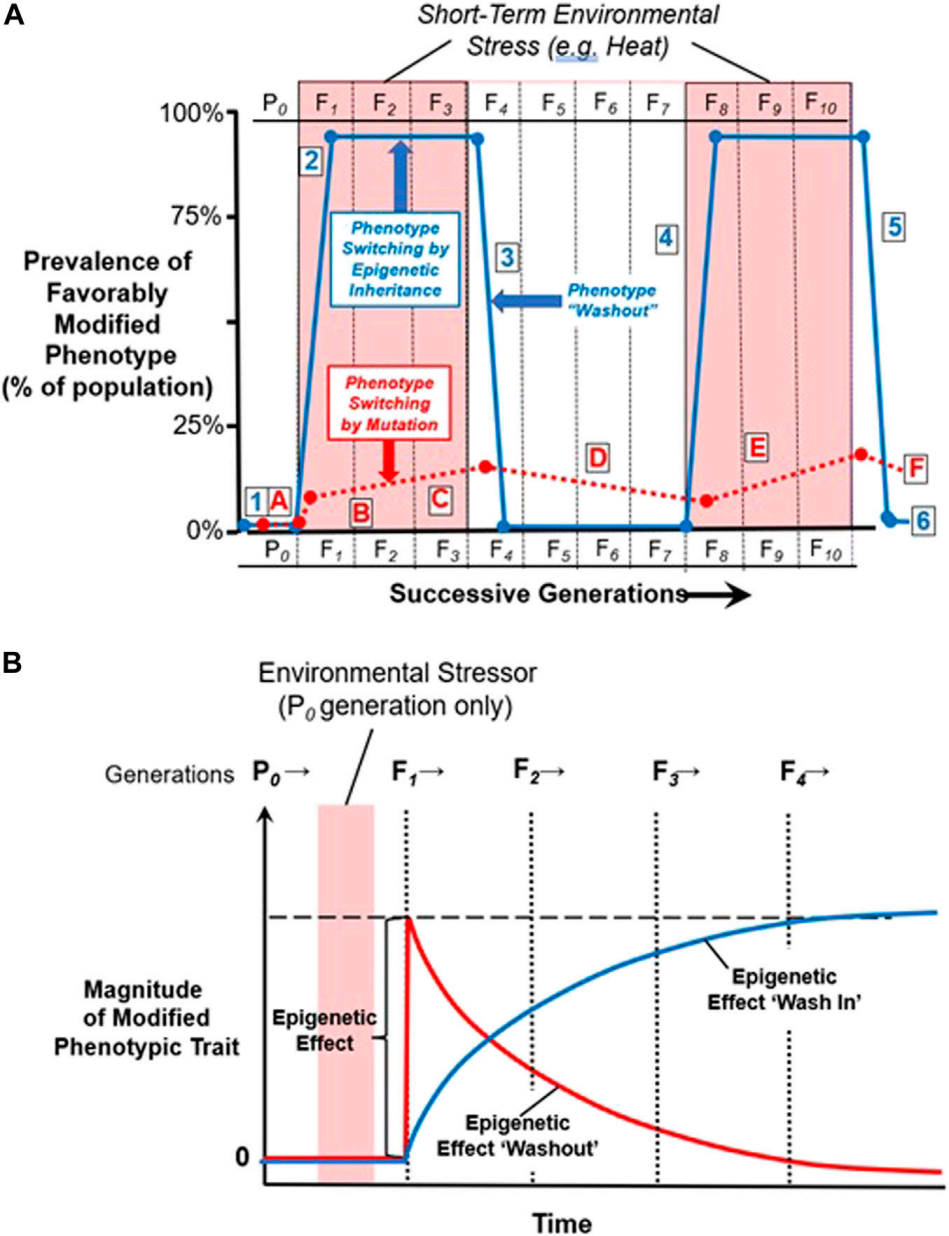
Phenotype switching. **(A)** Phenotypic switching by point mutation (A-F) vs. inheritance through effects of the epigenome on gene expression (1–6). Survival by point mutation (A-F): During mild environmental conditions, there is no selection pressure towards a modified phenotype with greater heat tolerance (A). Upon the appearance of elevated environmental temperature, in this example for three generations, there may be a strong selection pressure favoring favorable but rare point mutations that enhance heat tolerance. The very small proportion of individuals in the general population with this mutation are heavily selected for, but this mutation like most mutations only very slowly begins to be fixed in the general population (B-C, red dashed line). However, when elevated environmental temperature dissipates, this heat-tolerant mutation may confer no advantage and may even be detrimental under conditions of normal environmental temperature. Thus, individuals with this mutation may be selected against, resulting in slow decrease of this mutation in the general population (D). This cycle of positive and negative natural selection may be repeated with future alternating periods of environmental high temperature (E-F). Survival by epigenetic modification of gene expression–i.e., ‘epigenetic inheritance’ (1–6): During mild environmental conditions, there is no stimulus for changes in the epigenome that would create a modified phenotype with greater heat tolerance (1). Upon the appearance of elevated environmental temperature, however, there may be a change in pattern of epigenetic markers, resulting in changes in gene expression that confer enhanced heat tolerance. Importantly, this change in gene expression leading to greater survival in hot conditions is likely to occur in a large proportion of the population if not the entire population (2), unlike point mutations producing a heat tolerant phenotype that occur only in a few individuals at a time. This favorable phenotype produced by modified gene expression can continued to be epigenetically inherited across multiple generations as long as hot conditions exist. However, when elevated environmental temperature dissipates, the phenotype “washes out” as the epigenome and gene expression return to their normal configuration (3), and the heat-tolerant phenotype rapidly disappears in the general population. Just as for Mendelian inheritance, this cycle of epigenetic inheritance may be repeated (4–6) when another extended period of environmental high temperature returns (Adapted from Burggren (2016), licensed under CC BY 4.0). **(B)** Hypothetical changes in epigenetically modified phenotype over multiple generations. In this model, the epigenetically modified phenotype can arise fully in the F1 generation, and then fade out over subsequent generations (‘wash out’). Alternatively, the effect can appear in the F1 or even not until the F2 generation (‘wash in’), and then grow over subsequent generations before eventually fading (‘washout’). Empirical evidence supports both scenarios. Modified with permission from Burggren, (2015).

To emphasize, then, one of the key features of epigenetically-driven transgenerational inheritance is that it is rapidly reversible at both individual and population level. That is, whereas almost all of a population can simultaneously change under the influence of an environmentally-driven change in the epigenetic landscape, so too can almost all of the population revert back to the default phenotype, strictly driven by genotype, when the environmental stressor dissipates (Figure 6). Moreover, the epigenetically-modified phenotype can potentially disappear within a generation, or wash in or wash out over multiple generations (Figure 6B). It is this potential for reversal or ‘sunsetting’ of the temporarily modified phenotype that makes transgenerational epigenetic inheritance a potentially critical component in the assessment of the short- and long-term effects of climate change, as we will discuss below.

Finally, while we have emphasized the transient nature of epigenetically-induced phenotypic changes (certainly compared to evolution), such phenotypic changes can actually become fixed in the genome. While the mechanism is not fully understood, heavily methylated regions of DNA–specifically the CpG ‘islands’ within promoter regions of genes - are also highly mutagenic (Hodgkinson and Eyre-Walker, 2011; Xia et al., 2012; Behringer and Hall, 2015; Chen and Furano, 2015). Mutations of the promotors that become fixed can then permanently alter the phenotype, potentially resembling the transient phenotype that was epigenetically induced (Monk, 1995). Such mutations can, of course, be detrimental, neutral or occasionally adaptive, so natural selection then comes into play in determining the extent to which these mutations become fixed in a population. By extension, then there is the possibility that originally only temporary epigenetically-induced phenotypes providing tolerance to climate change-related stressors could take a ‘mutation shortcut’ through promoter mutation, leading to a new genome in individuals in a population experiencing climate change-related stress. If possible, this would represent the ‘best of both worlds’ regarding responses to climate change. Empirical evidence is currently lacking, however, and although the mechanisms for, and implications of, insertion into the genome of epigenetically induced phenotypes was proposed more than a quarter of a century ago (Monk, 1995), this research area remains a productive, if difficult, line of future research.

### 3.4 Adaptation through natural selection

Genetic changes in animal populations that produce a positive impact on fitness involve the process of adaptation through natural selection. The process of adaptation is so thoroughly understood and documented that we find it unnecessary to provide general references on the process of adaptation (but if we did it would naturally start with Darwin’s *On the Evolution of Species*). We do note, however, that adaptive evolution directly related to climate change occurs when population genotype frequencies change to express traits or phenotypes that provide increased fitness to new or increased environmental stressors (Bradshaw and Holzapfel, 2006; Bairos-Novak et al., 2021). In many species, however, a huge loss of fitness can occur as climate rapidly changes. For example, in North American pitcher-plant mosquitoes (*Wyeomyia smithii*), 88% of their fitness loss was due to experiencing incorrect seasonal cues that promoted genetic and phenotypic diversity (Bradshaw and Holzapfel, 2006). New genetic and consequently phenotypic changes are produced because the species are exposed to environmental conditions near or over their tolerance limits due to rapid climate change. These genetic changes in populations affect the timing of major life history events–e.g., when to develop, when to reproduce, when to enter dormancy, and when to migrate.

Phenotypic evolution producing tolerance or other responses to climate change depends on beneficial mutations that produce phenotypic variance, thus providing raw material for organisms to evolve adaptions for survival in changing environments. Species that live in stable environments typically show very slow rates of phenotypic evolution. Indeed, despite fascinating examples of evolution occurring in days, months or years, evolution is typically considered to be a process taking many hundreds, thousands or millions of years. When stacked up against the predicted rate of climate change of even the most stable of environments (e.g., the oceans), adaptation is not going to serve organisms well as they cope with climate change, let alone with weather. At the other extreme, organisms in unstable and/or rapidly changing environments often experience different selective agents and constraints compared to their ancestors, thus promoting rapid phenotypic evolution as they adapt genetically to their new environment (Lenski, 2001; Reusch and Wood, 2007; Hoffmann and Ross, 2018).

What is important to emphasize, then, is the rate of change associated with natural selection and climate change. The strength of selection pressure is a key driver of evolution rates. Heritability coefficient (‘narrow’ h^2^ or ‘broad’ H^2^) is a key parameter for predicting population responses in trait values for a single trait undergoing selection, thus allowing associated calculations of the relationship between the strength of selection pressures and rates of evolution. This coefficient has been used in a recent meta-analysis to analyze natural selection intensity on numerous traits in nearly 20 reef-building coral species across life stages/ages, growth forms, and environments (e.g., genotype-by-environment interactions) (Bairos-Novak et al., 2021). This analysis allowed evaluation of coral adaptation to climate change, which are undergoing strong selection for temperature tolerance due to anthropogenically driven increases in ocean temperatures. Reduced trait heritability was hypothesized, which would decrease environmental suitability and increase selective pressures, having as consequence a reduction in the capacity for populations to evolve in response to environmental change. Surprisingly, the analysis of Bairos-Novak et al. (2021) revealed that the heritability of coral traits has considerable heterogeneity that can be explained by differences between trait type. Traits such as gene expression have low heritability (h^2^ < 0.25). Moderate heritability (h^2^ = 0.25–0.5) was evident for photochemistry, growth, nutrient content, symbiont abundance, morphology, and symbiont community. Immune response, survival, and larval settlement success demonstrated the highest estimated heritability. An important life stage effect for certain trait type–heritability was detected. Thus, the estimated heritability for suffering bleaching was ∼9X higher in adults compared to juveniles, and ∼2X higher compared to larvae. Additionally, heritability of growth and nutrient content was ∼3X higher in adults than juveniles, and heritability of nutrient content was ∼4X greater in larvae *versus* adults. This example based on the responses of reef-building coral to ocean warming illustrates how quantitative measures of heritability are effective sensors for those traits under strong selection produced by climate change. This analysis also helped detect the most vulnerable life stages or populations and thus help predict the consequences of altering environmental factors.

Another factor in development that influences evolution of adaptations helping survival in a changing environment is simply life span and associated age of sexual maturity. An organism with a short life span–e.g., the brine shrimp *Artemia* reaching sexual maturity in ∼3 weeks after hatching–can show relatively rapid natural selection (Browne and Hoopes, 1990), which will act to fix favorable adaptations to rapidly changing environments. Contrast this with animals taking a long time to reach sexual maturity–e.g., the extreme example of the Greenland shark S*omniosus microcephalus*, estimated to require 150 years to reach sexual maturity (Nielsen et al., 2016). Put differently, a short life span simply allows for more generations (and thus more opportunities for natural selection) to be fitted into a given period of chronological time. Among factors contributing to this evolutionary race against time (and changing climate), life span deserves more consideration in future research.

One last note about rates of evolution through natural selection in a changing environment–especially a stochastically changing environment - is that individuals are “stuck” with the resulting adaptations that have become fixed in the genome of the population. If environmental conditions quickly reverse their direction of change, then natural selection must occur once again to ‘re-adapt’ the organism. As an example, consider species presumably in the early stages of adapting to the drought in the Southwestern United States during most of the 21st century. Thought to be the most severe drought in 1,200 years (Griffin and Anchukaitis, 2014; Williams et al., 2015), there were dire predictions of what was to come…. that is, until the winter of 2022–2023, in which especially California was lashed by winter rainstorm after rainstorm, resulting in a record snowpack in the Sierra Nevada Mountains^1^. The drought, which had especially seriously impacted water availability in California for the last 2 decades, was largely reversed in a matter of a few months. Hypothetically, species that through natural selection were beginning to skew towards drought tolerance over the more than 12 centuries of heavy selection pressure for water conservation–a suite of characters that was beginning to serve them well–potentially now could be non-adapted or even maladapted as wetter conditions returned to California in the spring of 2023.

### 3.5 Differentiating between mechanisms for phenotypic variation

As apparent from the discussion above, four major mechanisms can contribute–sometimes concurrently - to phenotypic variation in response to both short- and long-term environmental change. Designing experiments to identify and differentiate these mechanisms in particular individuals or populations can be problematic. Stochastic gene expression appears to be a fundamental component of gene expression in both eukaryotes and prokaryotes. Investigators have employed various techniques to assess stochastic gene expression and transcriptional bursting in particular and identify the associated molecular mechanisms: e.g., using single-molecule RNA single molecule fluorescence *in situ* hybridization (smRNA FISHFISH), using the MS2 green fluorescent protein to illuminate RNA stem loops by epifluorescence or confocal microscopy, and monitoring short-lived protein reporters allow the quantification of transcriptional bursting in particular.

Against this background of pervasive stochastic gene expression are changes in gene expression that are stimulated by environmental change interceding at the intracellular level. Sorting out stochastic from regulated gene expression can be challenging, as stochasticity has been implicated as an important process in producing the phenotypic variability upon which natural selection can act (Mineta et al., 2015). One approach could be to compare phenotypic variation in populations experiencing controlled environmental variation vs. those encountering no variation.

Determining phenotypic variation resulting from epigenetic vs. Mendelian inheritance has its own challenges. During most of the history of epigenetics, changes in DNA methylation, for example, could be correlated with changes in phenotype (Halabian et al., 2021), but there were no or only imperfect tools to actually experimentally alter epigenetic markers (Chandler and Jones, 1988). Thus, the equivalent of reverse genetics long available to the geneticist eluded epigeneticists. However, in the last decade new tools have emerged that allow the experimenter to actively alter epigenetic readers, writers, erasers and enhancers (Liu et al., 2016; Liu and Jaenisch, 2019; Zhang et al., 2020; Peng et al., 2022). Thus, the potential exists to compare phenotypic variability across multiple generations by manipulating epigenetic markers. A frequent criticism of studies of epigenetic inheritance as a source of phenotypic variation is that any altered phenotype in the F1 generation is simply the result of natural selection on a variable genotype for the P0, rather than different patterns of inherited epigenetic markers in the F1. While of limited availability, investigating clonal species (e.g., marbled crayfish) or strains of sexually reproducing species with very low heterozygosity (e.g., the NHGRI-1 strain of the wildtype zebrafish) can tell us much about the specific role of epigenetics by eliminating or at least minimizing natural selection as a factor in experiments on natural selection. Adding to the complexity of interpreting how epigenetic inheritance alters phenotypic plasticity is the fact that phenotypic variation from developmental phenotypic plasticity and epigenetic inheritance can readily co-exist. As described in Section 3.2.4, developmental programming from prenatal stress can act concurrently but separately through epigenetic or non-epigenetic modification of gene expression, and the resulting phenotypes that appear later in development can be similar, if not identical (Cao-Lei et al., 2020).

Finally, differentiation adaptation as a result of natural selection is not necessarily straightforward. Adaptation is like ‘beauty’—biologists recognize it when they see it. Perhaps surprisingly, there is no quantitative, universally accepted standard for measuring adaptation *per se* resulting from natural selection on phenotypic and genetic variation (Peck and Waxman, 2018; Cano et al., 2023). Effectiveness of adaptation has been derived from metrics as diverse as fitness measures (Brandon, 2014) to number of unique SNIPs (Moore, 2017) to a systematic overlapping outlier approach (Fraser and Whiting, 2020). If there is one certainty, adaptation occurs *across* generations, so any intragenerational phenotypic changes can most proximately be attributed to phenotypic plasticity (although it can and probably should be argued that the ability to be phenotypically plastic is, in of itself, a heritable trait!)

## 4 Can phenotype changes in developing and mature animals ‘catch up’ to climate change?

Having emphasized the different timelines and mechanisms by which phenotype can change, and having placed them against the backdrop of weather vs. climate, let us now turn to the matter at hand–exploring how organisms, especially developing organisms, react to climate change and whether these changes can be of sufficient speed to allow animals to survive, if not actually thrive extreme weather events.

### 4.1 Are phenotypic plasticity and epigenetic inheritance fast enough?

Whether individuals–developing or adult - can respond rapidly enough to be able to hold environmental challenges at bay, using any of the previously mentioned mechanisms, depends upon the rate at which the response can be mounted compared to the rate at which a ‘survival phenotype’ can be generated. A key question, then, is “*Can the biology of organisms change rapidly enough to avoid ecological death as a result of climate change?*” Many have considered this question (Somero, 2010; Bestion et al., 2015; Gunderson and Stillman, 2015; Sørensen et al., 2016; Buckley and Kingsolver, 2019; Fox et al., 2019; Huey and Buckley, 2022). The unequivocal answer is….maybe! It depends on the magnitude of the environmental change, and if it is in between of the individual tolerance limits, that means the speed response is directly related to the phenomics, proteomics, transcriptomics, or genomics present and needed on the environmental change event (Vulimiri et al., 2014). Continuing our focus on heat stress and tolerance, consider responses to increasing environmental temperatures. Animals have an upper temperature limit, formally described as Ct_max_ (Sørensen et al., 2016; Kingsolver and Umbanhowar, 2018; Lefevre et al., 2021). Above CT_max_ is a temperature zone of ecological death in which animals are non-functional, if not actually dying. Certainly, CT_max_ is subject to selection (Morgan et al., 2020; DeLiberto et al., 2022), with animals responding to long-term temperature stress in the form of higher average environmental temperatures (that is, ‘climate’) leading to an adaptation–in this instance, a higher CT_max_ embedded in the population. For example, such adaptations over multiple generations occurs in *Daphnia magna* (Cuenca Cambronero et al., 2018). At the same time, a shorter-term temperature increase (that is, ‘weather) can trigger epigenetic inheritance of a phenotype with higher CT_max_. And, of course, if the challenge of elevated temperature occurs during development or in adults, the physiological phenotype can change leading to higher CT_max_.

There are some empirical tests of whether thermal tolerance can change rapidly. Sørensen et al. (2016), studying thermal tolerance in *Drosophila melanogaster*, reported little plasticity of upper thermal limits and that this trait evolves rather slowly. Acclimation ability of *D. melanogaster* was only weakly correlated with environmental heterogeneity. These findings led Sørensen et al. (2016) to conclude that “*plasticity in upper thermal limits is unlikely to effectively buffer effects of global warming for species already close to their upper thermal boundaries*”. A meta-analysis examining thermal tolerance and acclimation in vertebrates and invertebrate taxa from marine, freshwater and terrestrial habitats arrived at similar conclusions (Gunderson and Stillman, 2015). Data collected from the clouded Sulphur Butterfly (*Colias eriphyle*) indicated that the slow rate of evolution in response to climate change–what the authors called “evolutionary lags” - results in greater sensitivity to climate and weather changes, but that phenotypic plasticity can reduce this tendency for evolutionary lags through facilitation of trait evolution (Buckley and Kingsolver, 2019). In a study on the common lizard (*Zootoca vivipara*) that combined both empirical data and modeling, it was predicted that temperate populations–often viewed as less sensitive to climate change than tropical ectotherm populations–were not immune to increased environmental temperatures. While surviving longer than tropical populations, temperate populations were nonetheless likely to face extinction (Bestion et al., 2015).

From these and similar studies, a mixed picture emerges. Phenotypic plasticity, acclimation and epigenetic inheritance (and even adaptation in animals with short life cycles) can all contribute to survival in the face of extreme weather generated by climate change, although determining the specific role of phenotypic plasticity in the evolution of favorable adaptations can be complex (Fox et al., 2019) as already discussed. Ultimately the effectiveness of changes in phenotype for ‘buffering’ climate change and especially extreme weather will likely be determined on a case-by-case basis–precluding broad conclusions about the effectiveness of various mechanisms for phenotypic plasticity. What is clear, however, is that the interplay of these mechanisms is likely to enable ‘bet hedging’ in the face of extreme weather associated with climate change, as we now explore.

### 4.2 Classic ‘bet-hedging’ against climate change

**FIGURE 7:**
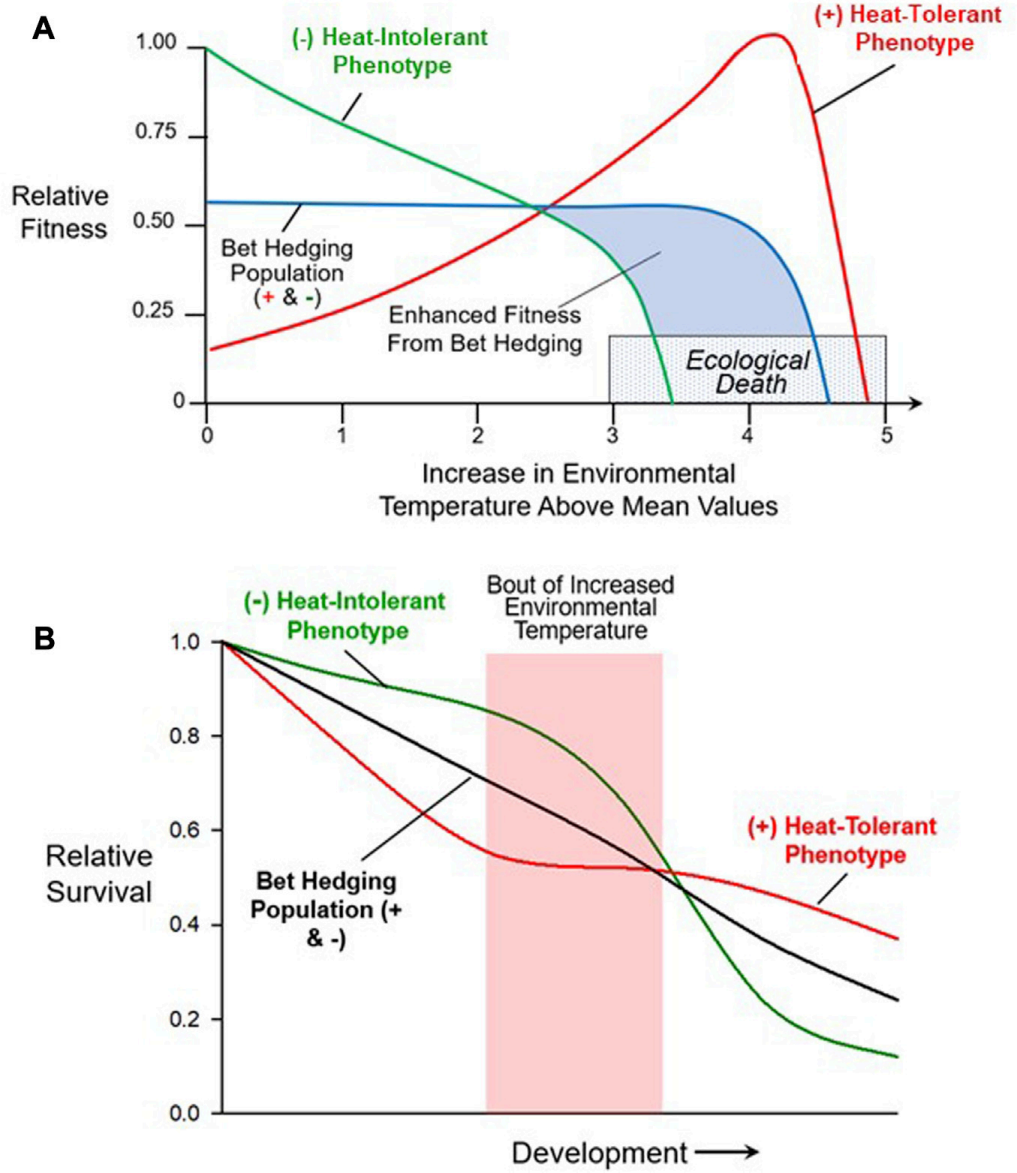
Bet hedging in a population through simultaneous multiple phenotypes. **(A)** This hypothetical example shows the changing relative fitness of three different populations as environmental temperature increases. The (−) population lacks any individuals with a heat tolerant phenotype. While its fitness in the absence of short-term temperature increases is higher, it experiences the greatest reduction in fitness as temperature increases above ‘normal’. The second population (+), possesses large numbers of individuals with the heat tolerant phenotype. Its overall fitness at lower temperatures is much lower than the (−) population lacking individuals with the heat tolerant phenotype, reflecting the heat-intolerant individuals elevated costs at these lower temperatures. However, as a consequence, this population with its heat tolerant individuals has a greater overall fitness as temperature increases up to a point at which no phenotype survives. This is an example of bet hedging in which the (+/−) population that has significant numbers of both heat tolerant (+) and heat intolerant (−) individuals has only intermediate fitness, but it will have some surviving individuals irrespective of whether temperature increases or stays constant. **(B)** This example shows how bet hedging can assist during development. As with example A), a heat-tolerant (+), heat-intolerant (−) and bet hedging population (+/−) are compared. All three populations decline in early development, reflecting natural mortality associated with the development process. Overall, however, the bet hedging population survives at a higher rate than the heat-intolerant population, but not as well as the heat-tolerant population. Thus, as in typical bet hedging, irrespective of whether there is a short-term temperature increase, the population with both phenotypes fairs better than the population without the (+) heat-tolerant phenotypic variation.

To determine the ‘dynamics of survival’ in the face of rapid changes due to extreme weather, it is important to differentiate between responses at the individual vs. population-level. Organisms use multiple adaptations^2^ to optimize fitness in the face of environmental variation (Haaland et al., 2019; Villa Martín et al., 2019; Zhou and Xue, 2022). One of these adaptations is phenotype ‘bet hedging’ at the population level (Hopper, 1999; Venable, 2007; Saether and Engen, 2015; Kreft et al., 2017). The classic view of bet hedging involves a population producing multiple phenotypes. These variable phenotypes in a population arise through two mechanisms–stochastic gene expression and phenotypic plasticity, as already discussed. Notably, and key to the success of bet hedging, is that some of these phenotypes will be less fit for the currently experienced environment, while others may have a higher fitness than the original population (Figure 7). That is, whatever the environmental stressor, there will always be some subset of the population better adapted for any encountered environment (within reason), reflected in either higher fitness (Figure 7A) or outright survival, especially during development and its periods of critical windows (Figure 7B). A commonly cited example involves trade-offs between a female animal laying large numbers of small eggs, or small numbers of large eggs. Typically, larger eggs are thought to convey extra resources to hatchlings, making each individual more resilient to environmental stressors, though this pattern is not universally observed (Olofsson et al., 2009). Such tradeoffs have been described in birds (Olsen et al., 2008), reptiles (Nussbaum, 1981), amphibians (Lips, 2001; Dziminski and Alford, 2005), fishes (Morrongiello et al., 2012; Shama, 2015) and a wide variety of invertebrates (Thomas and Poulin, 2003; Sørensen et al., 2016; Wang and Rogers, 2018).

Theoretical modeling combined with empirical evidence has long indicated that a mixture of both phenotypic plasticity and bet hedging *per se* is most likely to optimize fitness in large populations experiencing short-term environmental changes (Cohen, 1966; Philippi and Seger, 1989; Starrfelt and Kokko, 2012; Grantham et al., 2016). Bet hedging is not without cost, however. A population that is bet hedging will have lower overall fitness than one that does produce a wide variety of phenotypes, simply because some of these phenotypes will be mismatched to ‘normal’ conditions. However, during periods of environmental stress, these outlier phenotypes may be the only ones to survive. Of course, at some level of environmental stress, no phenotype may be suitable, and individuals will experience ecological death (the inability to reproduce), or actual death.

Much has been written about the bet hedging adaptation combined with phenotypic plasticity (see above) and both modeling and empirical evidence suggest that this approach can be effective–within reason. Combined with phenotypic plasticity, and developmental phenotypic plasticity in particular, changes in performance can certainly occur within a single generation, enhanced by transgenerational epigenetic inheritance. The concern is that these processes may be slower than human-induced climate change (Collins et al., 2013). This is especially true if one considers the stochastic elements of climate change manifesting in acute, extreme weather events. It is likely that many populations of organisms, if not whole species, will perish as a result of weather events before tolerant phenotypes can become representative of the species.

## 5 Discussion: what can physiologists do?

### 5.1 Some recommendations regarding future experimentation

We have emphasized that weather is somewhat unpredictable - as a variation on the old saying goes…. “*Don’t like the weather? Just wait a minute*!“. Consequently, we cannot make specific predictions about the short-, medium- and long-term viability of specific populations or species. What we can do, however, is begin to match our experimental paradigms to more closely resemble true environmental conditions. Currently, we rely heavily in our experimental design on artificial, step-wise changes. Instead, an approach using more realistic experimental conditions is increasingly being advocated, especially in the field of thermal biology (Angilletta, 2009; Bozinovic et al., 2011; Niehaus et al., 2012; Colinet et al., 2015; Czarnoleski et al., 2015; Dowd et al., 2015; Kingsolver et al., 2015; Dillon et al., 2016; Burggren, 2018; Morash et al., 2018; Burggren, 2019; Ridgway and Scott, 2023). Why is this change in protocol important? Data from the relatively few studies that feature stochastically varying environments indicate that there are significant differences in physiological performance in animals exposed to stochastically varying conditions compared to steady-state conditions (Saunders et al., 2002; Colinet et al., 2015; Drake et al., 2017; Ridgway and Scott, 2023). For example, in the fingered limpet (*Lottia digitalis*) exposed to steady state and fluctuating thermal environments, differences in thermal tolerance emerged between unpredictable trials using different heating and cooling patterns. In another invertebrate, the nematode *Heterakis gallinarium*, individuals exposed to a stochastically fluctuating thermal environment show significantly accelerated development compared to individuals experiencing a constant temperature reflecting the same overall thermal load (same ‘degree days’). Finally, in the killifish *F. heteroclitus*, the interaction between acclimation temperature and hypoxia tolerance was distinctly different between killifish exposed to fluctuating vs. constant temperature.

**FIGURE 8:**
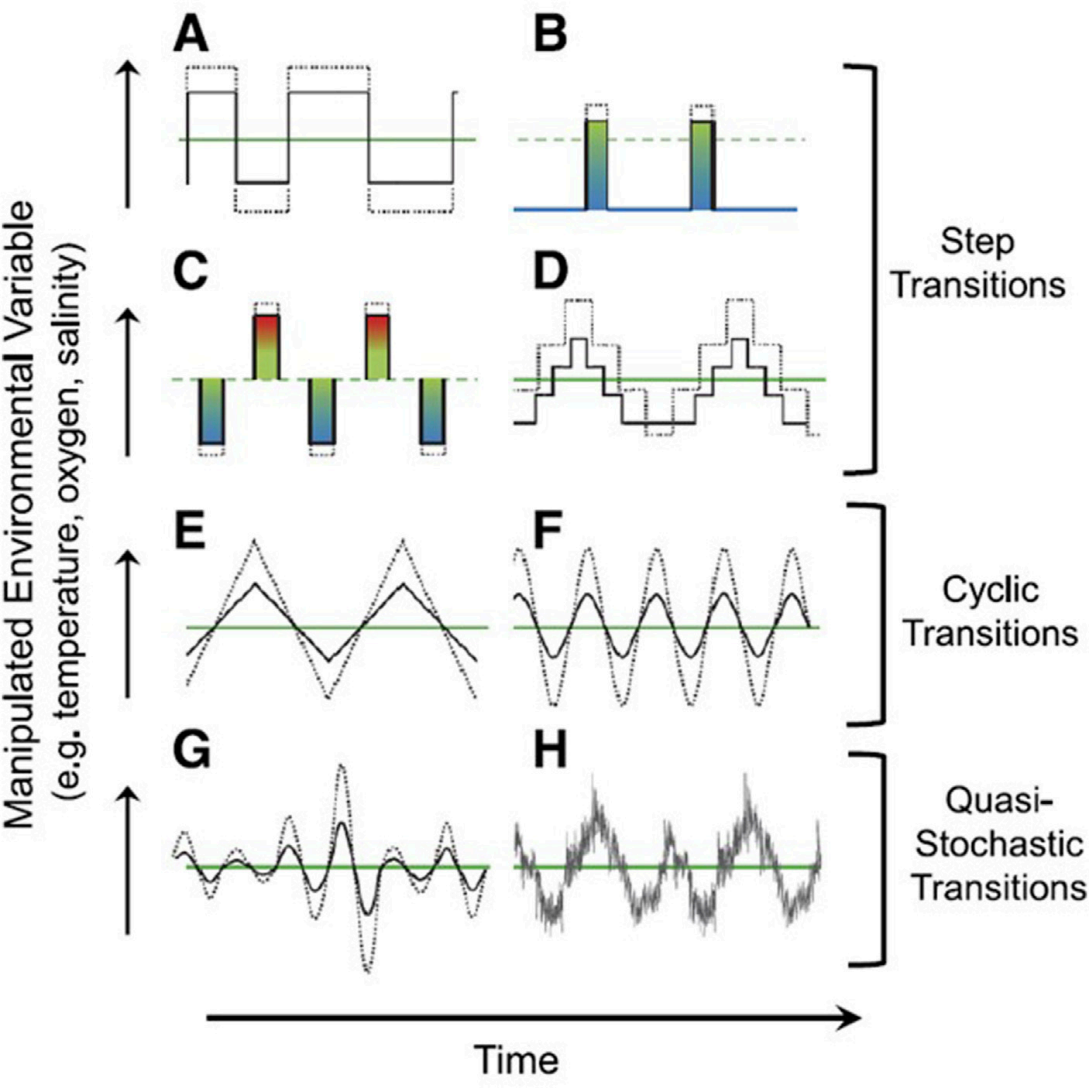
Experimental protocols in climate-related research that have been used to test biological responses to environmental challenges. **(A)** A simple step-wise change in conditions (e.g., from an acclimation temperature of 20°C to a new stable temperature 25°C) remains the most commonly employed protocol, in part because it is the most traditional, most easily interpreted and also the easiest to perform. **(B–D)** show variations on the theme of changing and environmental variable that begin to approach the complexity of natural environments. **(E, F)** indicate approaches with cyclic transitions, that can somewhat accurately represent the average temperature changes associated with diel, tidal or seasonal changes. **(G, H).** Imposing stochastic changes on cyclic or other protocols is simultaneously the most realistic (at least for terrestrial and small freshwater ecosystems), yet is also the most difficult to simulate. See text for further discussion. Reproduced from Burggren (2019), with permission from the American Physiological Society.

To show examples of various experimental protocols that could be employed in future studies, Figure 8 illustrates currently used experimental paradigms and less frequently employed (and more complex) paradigms that can help expand our as yet fragmentary understanding of how stochastic weather events can affect physiology as well other biological areas of study. While we suggest these protocols will be informative, being realistic we acknowledge that are not necessarily easy to implement. For example, environmental controllers are largely designed for stability rather than generating fluctuation, whether cyclical or stochastic. Fortunately, computerized control systems on a circuit board (e.g., Arduino, Teensy, LaunchPad, BBC Micro:bit and many others) are now available, inexpensive and easy to operate (Greenspan et al., 2016). Consequently, physiologists can use these and other technologies to produce acutely variable protocols that create changes in temperature, oxygen, pH or other variables and their combinations employed in experiments that more accurately reflect the real consequences of weather. Of course, having generated results from these experiments, the data must be statistically analyzed. Many conventional statistical approaches do not easily accommodate stochasticity of independent variables. Fortunately, techniques embracing stochasticity are available to physiologists (Awiszus, 1989; Coombes et al., 2004; Li and Li, 2009; Anwar et al., 2013; Yamanobe, 2013; Bressloff and Newby, 2014).

Another step towards experimental realism is to consider simulating not just a realistic stochastic change in a single environmental stressor, but incorporating multiple stressors that are varying independently or in concert (Relyea, 2004; Stillwell et al., 2007; Pandey et al., 2015; Westneat et al., 2019). There are likely to be synergistic or antagonistic effects between simultaneously occurring suites of stressors - the so-called “cocktail effect” (Esbaugh et al., 2018), including so-called “cross-protection” where exposure to one stressor enhances tolerance to a second stressor (Emri et al., 2022; Rodgers and Gomez Isaza, 2023).

Adding to the (necessary) complexity of understanding effects of weather/climate change, is showing how the effects of these combinations of stressors vary across developmental time. Experiments involving multiple combinations of multiple stressors at multiple points in development will not only be aided by the technology described above, but will likely require such technology, primarily because of the explosive growth in complexity as each variable and each degree of exposure is layered on. Nonetheless, it is in such experiments where true understanding exists of the nexus of phenotypic plasticity, environment and development.

Finally, *in silico* models with artificial intelligence capabilities will likely prove useful in informing future experiments. Certainly, the use of such models for studying not only consequences of climate but also predicting future effects have become key to understanding future species distribution (Hoban, 2014; Valladares et al., 2014; Eriksson and Rafajlović, 2022; Forester et al., 2023). *In silico* models of weather/climate effects have also become vital in agribusiness (Kumar, 2016; Vallejos et al., 2022; Liu et al., 2023).

Ideally, future experimental protocols will adopt variable fluctuation (if not actual stochasticity) and multiple simultaneously changed environmental variables, with the resulting data analyzed by a suite of statistical tools and used to produce AI-generated *in silico* models. Such studies will allow us to peel back the layers of the phenotypic plasticity ‘onion’, perhaps even revealing the combination of actual mechanisms by which organisms are attempting to cope with climate change-related extreme weather events.

### 5.2 Summary

We offer three general conclusions regarding the ability of phenotypic modification by various mechanisms (Figure 1), all combined in a bet hedging approach (Figure 7), to confer enhanced fitness of populations as climate change–in particular global warming–continues to progress.

• *Weather, climate and ecosystems.* Our experiments and their interpretations can best be performed if we develop a deeper appreciation of how different ecosystems experience different aspects of climate change, and global warming in particular. Yes, the global temperature will increase 2–4° by 2,100 (unless political winds take an unexpected shift), but that temperature range should not be used to inform experimental design for all organisms in all environments. Appreciating the complex and capricious nature of weather as a sub-component of climate will help us develop even better experiments as we go forward.
• *Multiple mechanisms for phenotype change.* As we have discussed, there are multiple mechanisms by which phenotypic changes can occur in individuals and populations: stochastic gene expression, phenotypic plasticity, transgenerational inheritance and adaptation. These, combined with bet hedging of the different mechanisms, can confer considerable capability for surviving weather events associated with climate change. However, while considerable focus is on developing animals (Figure 1A), we still have a large knowledge gap about the nexus of development and environment (see numerous chapters in (Burggren and Dubansky, 2018). This is especially the case because the vulnerability of the developing animal may be as or more important than the ability of the adult to reproduce. The phenotype of the adult does not matter if the developing animal never gets there (Burggren, 2018)!
• *Experimental approaches.* We suggest that physiologists develop control systems that are capable of simulating real-world situations and, with these systems, then determine the physiological responses of developing animals as they experience fluctuating conditions informed by weather and climate change. Data are emerging that indicate that the physiology we primarily know–that measured in steady-state conditions - may only be a subset of the total physiological repertoire of animals. To understand how animals will respond during development and as adults, we need to explore this ‘hidden physiology’.

Ultimately, the ‘unknowns’ greatly outweigh the ‘knowns’ with regard to how phenotype change in both developing and mature animals–ultimately leading to phenotype change through natural selection–will measure up to the demands of a changing climate.

## Footnotes

1 Mammoth Mountain, a popular ski resort in eastern central California, experienced more than 22 m of snowfall in the winter of 2022–2023!

2 We hesitate to use the word “*strategies*”, since organisms technically do not have strategies, but rather acquire favorable adaptations through natural selection.
